# Estimating the density of deep eutectic solvents applying supervised machine learning techniques

**DOI:** 10.1038/s41598-022-08842-5

**Published:** 2022-03-23

**Authors:** Mohammadjavad Abdollahzadeh, Marzieh Khosravi, Behnam Hajipour Khire Masjidi, Amin Samimi Behbahan, Ali Bagherzadeh, Amir Shahkar, Farzad Tat Shahdost

**Affiliations:** 1 School of Mechanical Engineering, Center of Excellence in Energy Conversion (CEEC), Sharif University of Technology, Azadi Avenue, P. O. Box 11365-9567, Tehran, Iran; 2 Department of Civil and Environmental Engineering, College of Engineering, Villanova University, Pennsylvania, USA; 3 Computer Engineering Department, Tehran North Branch, Islamic Azad University, Tehran, Iran; 4 Department of Mechanical Engineering, Behbahan Khatam Alanbia University of Technology, Behbahan, Iran; 5 Department of Mechanical Engineering and Mechanics, Drexel University, Philadelphia, PA 19104 USA; 6 Department of Transportation Engineering, Karadeniz Technical University, Trabzon, 61080 Turkey; 7 Department of Electrical Engineering, Garmsar Branch, Islamic Azad University, Semnan, Iran

**Keywords:** Chemical engineering, Materials chemistry

## Abstract

Deep eutectic solvents (DES) are recently synthesized to cover limitations of conventional solvents. These green solvents have wide ranges of potential usages in real-life applications. Precise measuring or accurate estimating thermophysical properties of DESs is a prerequisite for their successful applications. Density is likely the most crucial affecting characteristic on the solvation ability of DESs. This study utilizes seven machine learning techniques to estimate the density of 149 deep eutectic solvents. The density is anticipated as a function of temperature, critical pressure and temperature, and acentric factor. The LSSVR (least-squares support vector regression) presents the highest accuracy among 1530 constructed intelligent estimators. The LSSVR predicts 1239 densities with the mean absolute percentage error (MAPE) of 0.26% and R^2^ = 0.99798. Comparing the LSSVR and four empirical correlations revealed that the earlier possesses the highest accuracy level. The prediction accuracy of the LSSVR (i.e., MAPE = 0. 26%) is 74.5% better than the best-obtained results by the empirical correlations (i.e., MAPE = 1.02%).

## Introduction

The separation-based equipment has always been an accompanied part of chemical processes^1^, pharmaceutical industries^2^, water/wastewater treatment processes^3–5^, and environmental protection^6^. The separation process primary responsibility is to remove contaminants from feed stocks and effluent liquid and gas streams, purify final products, and recover unreacted materials^7^. Although diverse separation processes have already been established, the solvent-based technique has a wider range of applications.

The green chemistry principles introduced by Anastas and Warner suggested constructing chemical processes that eliminate or at least reduce utilizing/generating harmful substances^8^. Since the traditional organic solvents undesirably impact the ecosystem and human health, researchers have paid great attention to synthesizing green, sustainable, and environmentally-friendly solvents. Attempts to fulfill this objective have resulted in suggesting supercritical fluids^9^, renewable solvents^10^, liquid polymers^11^, and ionic liquids^12^.

Deep eutectic solvents (DES) have recently been recommended as materials that have the favorable features of ionic liquids and cover their undesirable characteristics^13–16^. Deep eutectic solvents have readily been synthesized by mixing two main agents, i.e., hydrogen bond donor (HBD) and hydrogen bond acceptor (HBA)^17^. Hydrogen bond formations between HBA and HBD resulted in synthesizing a mixture with a melting point highly smaller than its ingredient^17^. Generally, deep eutectic solvents are biodegradable, inexpensive, non-toxic, non-volatile, thermally/chemically stable, and easy to manufacture^17^.

Despite a short life of deep eutectic solvents, they have engaged in diverse applications, including material synthesis^18^, separation processes^13^, nanotechnology^19^, environmental protection^17^, biotechnology^20^, and pharmaceutical processing^21^.

The accurate values of volumetric properties of deep eutectic solvents, like density, are essential for feasibility study and detailed design of any possible industrial usages of DESs^22^. Moreover, selecting an appropriate DES with the desired density is an arduous task to be accomplished through laboratory-scale investigations.

Therefore, constructing a predictive tool to anticipate the density of deep eutectic solvents may be helpful in this regard. Although a few empirical correlations have technically been built for estimating the density of DESs (see Sect. 3.1), to the best of our knowledge, no intelligent scheme has been suggested yet. Hence, this work utilizes five intelligent schemes for calculating the DES’s density from some available variables, i.e., temperature and DES’s inherent features (acentric factor and critical pressure and temperature) and compares their prediction accuracy. This is the most comprehensive modeling study yet conducted for mechanizing the DES’s characterization. The databank includes massive laboratory-scale density measurements gathered from the literature to certify that the suggested paradigms are general and robust. The reliability of the constructed intelligent estimators is higher than the other correlations proposed in the literature.

## Laboratory-measured datasets

The objective of the current study is constructing an intelligent tool to approximate the density of deep eutectic solvents precisely. Identical to the regression-based correlation^23^, all intelligent methods also need a laboratory-measured database to adjust their parameters and test their prediction reliability^24,25^. Thus, 1239 experimentally measured datasets for the density of deep eutectic solvents have been gathered from thirty references and engaged in the model development/validation stage. The summary of the collected density data has been presented in Table 1. This table introduces the name of hydrogen bond donors and hydrogen bond acceptors of the considered deep eutectic solvents. As Table 1 shows, the gathered databank includes thirteen HBA and forty-two HBD ingredients. This table also indicates the number of measurements and ranges of the working temperature and measured density.

**Table 1:** Summary of the reported laboratory-measured density for diverse deep eutectic solvents in the literature.

HBA agent	HBD agent	Temperature range (K)	Density range (kg/m^3^)	Numbers of data	References
Acetyl choline chloride (HBA #1)	1,2,4-triazole, D-Fructose, D-Glucose, D-Mannose, D-Ribose, D-Xylose, Guaiacol, Imidazole, Levulinic acid	293.15–363.15	1089.5–1275.0	120	^13–16^
Allyl triphenylphosphonium bromide (HBA #2)	Diethylene glycol, Triethylene glycol	293.15–343.15	1108.4–1201.1	66	^17^
Benzyl tripropyl ammonium Chloride (HBA #3)	Ethylene glycol, Glycol, Lactic acid, Oxalic acid, Phenol	293.15–348.15	1027.6–1263.0	56	^30^
Betaine (HBA #4)	Lactic acid, Levulinic acid	293.15–343.15	1126.6–1208.9	32	^31^
Benzyldimethyl (2-hydroxyethyl) ammonium chloride (HBA #5)	D-Fructose, D-Glucose, D-Mannose, D-Ribose, D-Xylose	293.15–353.15	1192.0–1262.0	65	^14^
Choline chloride (HBA #6)	1,2-propanediol, 1,4-butanediol, 2,3-butanediol, Acetamide, Citric acid, D-Fructose, D-Glucose, D-Mannose, D-Ribose, D-Sorbitol, D-Sucrose, D-Xylose, Ethylene glycol, Glycolic, Glycolic acid, Guaiacol, Levulinic acid, Malonic acid, N-furfuryl alcohol, O-Cresol, Oxalic acid, P-Chlorophenol, P-Cresol, Phenol, p-Toluenesulfonic acid, Tartaric acid, Triethylene glycol, Urea, Xylitol	283.15–368.15	1019.7–1350.0	439	^14–16,19,32–48^
Diethylamine hydrochloride (HBA #7)	Guaiacol	293.15–323.15	1075.8–1106.1	12	^15^
L-proline (HBA #8)	Lactic acid, Levulinic acid	293.15–343.15	1164.0–1265.1	22	^31^
Methyl triphenylphosphonium bromide (HBA #9)	Ethylene glycol, Glycerol	298.15–368.15	1168.8–1306.4	105	^47,49^
N, N diethylenethanol ammonium chloride (HBA #10)	Ethylene glycol, Glycerol	298.15–368.15	1054.6–1220.1	110	^33,47^
Tetrabutylammonium chloride (HBA #11)	Arginine, Aspartic acid, Ethylene glycol, Glutamic acid, Glycerol, Phenylacetic acid, Propionic acid, Triethylene glycol, Levulinic acid	288.15–353.15	928.0–1154.0	158	^16,17,50,51^
Tetraethylammonium bromide (HBA #12)	Levulinic acid, Ethylene glycol, Glycerol	293.15–343.15	975.7–1177.4	33	^16,52^
Trimethylglicine (HBA #13)	2-Chloro benzoic acid, Benzoic acid, Mandelic acid, Phenylacetic acid	298.15–373.15	1110.0–1290.0	21	^53^

### Critical pressure, critical temperature, and acentric factor

This study aims to build a single model to anticipate the density of 149 various deep eutectic solvents. Therefore, it is mandatory to include inherent characteristics of these materials in the list of independent variables. The three-parameter corresponding state theory explains that each material has its own specific acentric factor, critical temperature, and critical pressure^26^. Hence, these parameters could help the machine learning method distinguish different deep eutectic solvents and discriminate among their density values^27^. Haghbakhsh et al.^28^ utilized the improved Lydersen-Joback–Reid group contribution^12^ and the Lee-Kesler mixing rules^29^ to estimate acentric factor and critical temperature/pressure of different deep eutectic solvents.

Table 2 presents the range of these inherent characteristics for all considered deep eutectic solvents^28^. The supplementary excel files includes all experimental databank utilized in the current study.

**Table 2:** The reported critical pressure, critical temperature, and acentric factor for the considered deep eutectic solvents^28^.

HBA agent	HBD agent	Range of Tc (K)	Range of Pc (MPa)	Range of ω (-)
Acetylcholine chloride	1,2,4-triazole, D-Fructose, D-Glucose, D-Mannose, D-Ribose, D-Xylose, Guaiacol, Imidazole, Levulinic acid	635.41–846.26	2.3489–4.7708	0.4154–1.4097
Allyl triphenylphosphonium bromide	Diethylene glycol, Triethylene glycol	696.23–817.24	2.5162–3.4033	0.9700–1.0819
Benzyl tripropyl ammonium chloride	Ethylene glycol, Glycol, Lactic acid, Oxalic acid, Phenol	644.10–744.01	2.7293–3.7822	0.5152–1.2862
Betaine	Lactic acid, Levulinic acid	668.50–701.24	3.8938–4.7230	0.6195–0.8755
Benzyldimethyl (2-hydroxyethyl) ammonium chloride	D-Fructose, D-Glucose, D-Mannose, D-Ribose, D-Xylose	843.35–908.27	2.1009–2.4724	1.3871–1.5684
Choline chloride	1,2-propanediol, 1,4-butanediol, 2,3-butanediol, Acetamide, Citric acid, D-Fructose, D-Glucose, D-Mannose, D-Ribose, D-Sorbitol, D-Sucrose, D-Xylose, Ethylene glycol, Glycolic, Glycolic acid, Guaiacol, Levulinic acid, Malonic acid, N-furfuryl alcohol, O-Cresol, Oxalic acid, P-Chlorophenol, P-Cresol, Phenol, p-Toluenesulfonic acid, Tartaric acid, Triethylene glycol, Urea, Xylitol	600.98–1084.19	2.4301–5.2851	0.4770–1.5011
Diethylamine hydrochloride	Guaiacol	680.55–694.99	4.3476–4.5308	0.4659–0.4737
L-proline	Lactic acid, Levulinic acid	721.95–745.61	4.2880–4.8538	0.7044–0.8243
Methyl triphenylphosphonium bromide	Ethylene glycol, Glycerol	666.50–843.78	2.8329–4.2132	0.9031–1.2929
N, N diethylenethanol ammonium chloride	Ethylene glycol, Glycerol	604.66–699.38	3.2396–4.4874	0.9195–1.3207
Tetrabutylammonium chloride	Arginine, Aspartic acid, Ethylene glycol, Glutamic acid, Glycerol, Phenylacetic acid, Propionic acid, Triethylene glycol, Levulinic acid	588.75–808.05	1.4380–4.1852	0.6212–1.3576
Tetraethylammonium bromide	Levulinic acid, Ethylene glycol, Glycerol	687.77–793.24	1.9639–2.8859	0.6275–1.3155
Trimethylglycine	2-Chloro benzoic acid, Benzoic acid, Mandelic acid, Phenylacetic acid	711.92–780.73	3.5488–4.0881	0.5609–0.8301

In order to reduce the table size, the reported values have been presented for deep eutectic solvents based on their hydrogen bond acceptor type. Specific values of the acentric factor, critical temperature, and critical pressure for each deep eutectic solvent can be found in Haghbakhsh et al. article^28^.

## Estimation scenarios for density of deep eutectic solvents

The literature has suggested several empirical correlations for estimating the liquid’s density. Furthermore, the current study focuses on seven machine learning methods to anticipate the density of 149 deep eutectic solvents. The mathematical formulation/background of the available empirical correlations and machine learning methods has been briefly reviewed in this section.

### Empirical correlations

#### Rackett correlation

Rackett’s correlation is likely the first equation developed to calculate the saturated liquid’s density^54^. As Eq. (1) explains, the molar volume ($\nu$) is estimated as a function of temperature (*T*) and critical pressure (*Pc*), molar volume ($\nu_{c}$), and temperature (*Tc*). *R* and *Tr* show the gas constant and reduced temperature (Eq. 2), respectively.

1 $$\nu = \left( {RT_{c} /P_{c} } \right)\left( {P_{c} \nu_{c} /RT_{c} } \right)^{{1 + \left( {1 - T_{r} } \right)^{0.2857} }}$$

2 $$T_{r} = T/T_{c}$$

Equation (3) is then possible to be used to reach the density ($\rho$) from the molecular weight (*M*) and estimated molar volume.

3 $$\rho = M/\nu$$

Although Rackett’s correlation was initially suggested for the saturated liquid’s density, it has also presented good predictions for the deep eutectic solvent^55^.

#### Spencer and Danner correlation

Spencer and Danner incorporate a base molar volume measurement ($\nu_{ref}$) at a base temperature ($T_{ref}$) in Rackett’s correlation^56^. Equations (4) and (5) introduce the modified Rackett model, i.e., the Spencer and Danner correlation.

4 $$\nu = \nu_{ref} Z^{{\left( {1 - T_{r} } \right)^{{0.2857 - \left[ {1 - \left( {T_{ref} /T_{c} } \right)} \right]^{0.2857} }} }}$$

5 $$Z = \left( {\nu_{ref} P_{c} /RT_{c} } \right)^{{\left[ {1 + \left( {1 - \left( {T_{ref} /T_{c} } \right)} \right)^{0.2857} } \right]^{ - 1} }}$$

#### Mjalli et al. correlation

Mjalli et al.^57^ suggested a technical correlation for the density of deep eutectic solvents by reformulating the Spencer and Danner model. Equations (6) and (7) express the mathematical shape of the developed correlation by Mjalli et al.^57^.

6 $$\nu = \nu_{ref} Z^{{\left( {1 - T_{r} } \right)^{{2.2857 - \left[ {1 - \left( {T_{ref} /T_{c} } \right)} \right]^{2.2857} }} }}$$

7 $$Z = \left( {\nu_{ref} P_{c} /RT_{c} } \right)^{{\left[ {0.2083 + \left( {T_{ref} /T_{c} } \right)} \right]^{2.2857} }}$$

#### Haghbakhsh et al. correlation

Haghbakhsh et al. recently proposed a correlation for calculating the density of deep eutectic solvents from the working and critical temperatures, acentric factor (ω), and critical molar volume^28^.

8 $$\rho = \alpha - 4.64 \times 10^{ - 4} \times T$$

9 $$\alpha = - 1.13 \times 10^{ - 6} \times T_{c}^{2} + 2.566 \times 10^{ - 3} \times T_{c} + 0.2376 \times \omega^{0.2211} - 4.67 \times 10^{ - 4} \times \nu_{c}$$

It can be seen that all empirical correlations utilize the temperature and inherent characteristics of the material (a combination of the ν_c_, P_c_, T_c_, and ω) to formulize the liquid’s density. Since the first three inherent properties (T_c_, P_c_, and ν_c_) are related through the following equation, it is unnecessary to utilize all of them.

10 $$P_{c} \times \nu_{c} = R \times T_{c}$$

Therefore, the current study only utilizes temperature, T_c_, P_c_, and ω to estimate the DES’s density employing different intelligent estimators (Eq. 11).

11 $$\rho_{pred}^{DES} = f\left( {T,P_{c}^{DES} ,T_{c}^{DES} ,\omega^{DES} } \right)$$

### Computational intelligent methods

Wide ranges of supervised and unsupervised artificial intelligence techniques have been suggested and applied in different modeling studies^58–63^. The working procedures of the used machine learning methods, i.e., least-squares support vector regression (LSSVR), hybrid neuro-fuzzy system, and five types of artificial neural networks have been briefly explained in this section.

#### Least-squares support vector regression

This intelligent estimator employs a particular equation (i.e., linear, Gaussian, and polynomial kernel function) to transfer original independent variables ($\xi$) to a multi-dimensional computational domain. The following equation defines these functions.

12 $$\varphi \left( {\xi_{i} ,\xi_{j} } \right) = \left\{ {\begin{array}{*{20}l} {\xi_{i}^{T} \xi_{j} } \hfill & {Linear} \hfill \\ {\left( {\xi_{i}^{T} \xi_{j} +\varepsilon } \right)^{\sigma } } \hfill & {Polynomial} \hfill \\ {\exp \left( { - \left\| {\xi_{i} - \xi_{j} } \right\|^{2} /2\delta^{2} } \right)} \hfill & {Gaussian} \hfill \\ \end{array} } \right.$$

The superscript of *T* shows the transpose operation. In addition, $\varepsilon$, $\sigma$, and $\delta$ are the kernel-related parameters.

It is then possible to linearly relate the dependent ($\gamma$) to the independent ($\chi$) variables in this new computational domain utilizing Eq. (13).

13 $$\gamma_{LSSVR} (\chi ) = w^{T} \varphi \left( \chi \right) + b$$

In Eq. (13), $\gamma_{LSSVR}$ represents the estimated target by the least-squares support vector regression. Furthermore, *w* and *b* are adjustable coefficients of this intelligent model. In summary, the kernel type is the main topology feature of the LSSVR that should be determined by a practical scenario like the trial-and-error process^64^.

The detailed working process of the least-squares support vector machine has recently been explained by Nabavi et al.^64^.

#### Artificial neural networks

This neuron-based machine learning method is the most widely-used tool as either estimator^65,66^ or classifier^67^. The working process of the artificial neural network is handled by a combination of linear (LPart) and non-linear (NLPart) operations conducted by the neuron as follows^68^:

14 $$LPart = \vec{w}\vec{\xi } + b$$

15 $$NLPart = \phi \left( {\vec{w}\vec{\xi } + b} \right)$$

*w*, *b*, and $\phi$ are weight and bias coefficients and activation function, respectively. Although a linear activation function exists, the non-linear, continuous, and differentiable ones often provide artificial neural networks with a better generalization ability^69^. Equation (16) defines several widely-used activation functions in the field of artificial neural networks.

16 $$\phi \left( {LPart} \right) = \left\{ {\begin{array}{*{20}l} {LPart} \hfill & {Linear} \hfill \\ {\frac{1}{{1 + \exp \left( { - LPart} \right)}}} \hfill & {Logarithm\,sigmoid} \hfill \\ {\frac{2}{{1 + \exp \left( { - 2\, \times LPart} \right)}} - 1} \hfill & {Tangent\,sigmoid} \hfill \\ {\exp \left( { - LPart^{2} /2\delta^{2} } \right)} \hfill & {Gaussian} \hfill \\ \end{array} } \right.$$

Different artificial neural networks can be built by inserting neurons in several successive neuronic layers. The multilayer perceptron^70^, recurrent^71–73^, cascade feedforward^70^, radial basis function^70^, and general regression^74^ neural networks are those neuron-based estimators utilized in the current study. Interested readers are referred to the book written by Hagan et al. for the detailed understanding of the working procedure of these artificial neural networks^75^.

#### Hybrid neuro-fuzzy systems

The idea of combining the artificial neural network^76,77^ and fuzzy logic^78,79^ has resulted in a new class of machine learning, namely adaptive neuro-fuzzy inference system^80,81^. This method estimates a target response employing five successive layers (i.e., fuzzification, rule, normalization, defuzzification, and output)^82^. Shojaei et al. have comprehensively described the mathematical operations performed in each layer of the adaptive neuro-fuzzy inference system^82^. The membership function utilized in the fuzzification layer^83^, numbers of the cluster^80^, cluster radius^84^, and training algorithm^25^ are the main structural features that are often regulated by the trial-and-error scenario.

## Results and discussions

This section comprehensively explains the followed procedure to choose the best intelligent method for estimating the DES’s density and determining its structural features. The accuracy of this smart approach and available correlations in the literature has then been compared. Several numerical and graphical analyses have also been employed for further monitoring the accuracy of the best model for predicting the density of deep eutectic solvents.

### Constructing intelligent models

#### Topology determination

The topology of machine learning methods is often determined by trial-and-error practice^85–87^. This practical scenario changes the core features of a machine learning scheme and monitors its accuracy in diverse stages of the model development^88–90^. Table 3 specifies the core features of the considered intelligent techniques and their investigation range during the trial-and-error procedure. The literature approved that artificial neural networks with one hidden layer are accurate enough to simulate a wide range of problems^72,91–93^. Consequently, the multilayer perceptron (MLP), recurrent (RNN), cascade feedforward (CFF), general regression (GR), and radial basis function (RBF) have been fabricated with only one hidden layer.

**Table 3:** The name and range of deciding features of each intelligent estimator during the trial-and-error process.

Model name	Deciding features changed during the trial-and-error process	Numbers of model
LSSVR	Types of the kernel function, i.e., linear, polynomial, and Gaussian	210
MLP	Numbers of the hidden neuron, i.e., 1, 2, …, 11	220
CFF	Numbers of the hidden neuron, i.e., 1, 2, …, 10	200
GR	Spread values of the Gaussian activation function, i.e., 1 × 10^–6^, …, 10	220
RBF	Numbers of the hidden neuron, i.e., 1, 2, …, 11 Spread values of the Gaussian activation function, i.e., 1 × 10^–6^, …, 10	220
RNN	Numbers of the hidden neuron, i.e., 1, 2, …, 6	180
ANFIS	Types of membership function, i.e., subtractive and c-mean clustering Numbers of the cluster, i.e., 2,3, …, 12 Values of the cluster radius, i.e., 0.5, 0.53571, …, 1 Training algorithm, i.e., hybrid and backpropagation	360

#### Selecting the best topology of the intelligent methods

The core features of the machine learning methods have been changed according to the reported values in Table 3, both training and testing stages have been performed, and accuracy has been monitored utilizing several statistical indexes. Various uncertainty criteria, including MAPE (mean absolute percentage error), RMSE (root mean square error), RAPE (relative absolute percentage error), MAE (mean absolute error), and R^2^ (regression coefficient), have been utilized to accuracy monitor of the developed intelligent scenarios and selecting the most precise ones.

Equations (17) to (21) express the mathematical shapes of the MAPE, MAE, RAE, RMSE, and R^2^, respectively.

17 $$MAPE\% = \left( {100/n} \right) \times \sum\limits_{i = 1}^{n} {\left( {\left| {\rho_{\exp } - \rho_{pred} } \right|/\rho_{\exp } } \right)}_{i}$$

18 $$MAE = \left( {1/n} \right) \times \sum\limits_{i = 1}^{n} {\left| {\rho_{\exp } - \rho_{pred} } \right|}_{i}$$

19 $$RAPE\% = 100 \times \sum\limits_{i = 1}^{n} {\left| {\rho_{\exp } - \rho_{pred} } \right|_{i} /\sum\limits_{j = 1}^{n} {\left| {\rho_{\exp } - \rho_{\exp }^{ave} } \right|_{i} } }$$

20 $$RMSE = \sqrt {\sum\limits_{i = 1}^{n} {\left( {\rho_{\exp } - \rho_{pred} } \right)}_{i}^{2} /n}$$

21 $$R^{2} = 1 - \left\{ {\sum\limits_{i = 1}^{n} {\left( {\rho_{\exp } - \rho_{pred} } \right)_{i}^{2} /\sum\limits_{i = 1}^{n} {\left( {\rho_{\exp } - \rho_{\exp }^{ave} } \right)_{i}^{2} } } } \right\}$$

These equations only need the actual ($\rho_{\exp }$), predicted ($\rho_{pred}$), and average ($\rho_{\exp }^{ave}$) density values and numbers of the dataset (*n*) to measure the accuracy of any constructed model.

The most precise density estimations obtained by each machine learning method have been reported in Table 4. The accuracy monitoring approves that 1) the Gaussian function is the best kernel for LSSVR, 2) eleven hidden neurons is the best feature for the MLP, 3) ten hidden neurons provides the CFF with the best performance, 4) spread factor of 0.04312 and 1053 hidden neurons should be used in the GR structure, 5) the RBF is better to construct by spread factor of 1.0526 and eleven hidden neurons, and 6) the ANFIS (adaptive neuro-fuzzy inference systems) with the subtractive clustering membership function, twelve clusters, and hybrid training algorithm has the best performance.

**Table 4:** The most precise prediction obtained by different intelligent estimators (1053 training and 186 testing datasets).

Model name	Datasets	MAPE%	MAE	RAPE%	RMSE	R^2^
LSSVR	Training data	0.25	2.86	3.94	5.64	0.99799
	Testing data	0.30	3.38	4.75	5.68	0.99794
	Training + Testing	0.26	2.94	4.06	5.65	0.99798
MLP	Training data	1.04	11.75	16.54	18.16	0.97805
	Testing data	1.10	12.47	15.68	19.98	0.97801
	Training + Testing	1.05	11.86	16.39	18.44	0.97804
CFF	Training data	1.16	13.29	18.12	18.53	0.97844
	Testing data	1.16	13.20	19.88	18.61	0.97345
	Training + Testing	1.16	13.28	18.36	18.54	0.97780
GR	Training data	0.95	10.73	14.82	16.92	0.98246
	Testing data	1.52	17.10	23.72	27.70	0.94916
	Training + Testing	1.04	11.68	16.16	18.94	0.97758
RBF	Training data	2.98	33.87	46.13	44.17	0.86954
	Testing data	2.56	29.32	44.33	38.72	0.88919
	Training + Testing	2.92	33.18	45.88	43.39	0.87158
RNN	Training data	2.52	28.57	39.49	36.93	0.90923
	Testing data	2.58	28.95	40.21	39.10	0.89494
	Training + Testing	2.53	28.63	39.59	37.26	0.90701
ANFIS	Training data	1.17	13.40	18.61	19.22	0.97605
	Testing data	1.21	13.89	18.76	20.23	0.97402
	Training + Testing	1.17	13.47	18.63	19.37	0.97573

Although all these prediction accuracies confirm a high level of consistency with the laboratory-measured density, the LSSVR and RBF neural network present the highest and lowest precise results, respectively. For systematical approving this claim, the subsequent analysis has ranked these selected intelligent models based on their prediction accuracy in different stages of model development.

#### Selecting the best intelligent model using the ranking analysis

The ranking analysis is a well-established procedure to arrange several models based on their performance. The previous step measured the prediction ability of the seven selected intelligent models using five well-known statistical indexes. Now the ranking analysis utilizes the numerical values of these statistical indexes to arrange them from the best to the worst model. Equation (22) indicates that the selected models have been ranked based on their average rankings over five statistical criteria (*indx*).

22 $$Rank = round\left( {\sum\limits_{indx = 1}^{5} {Rank_{indx} } /5} \right)$$

**Figure 1:**
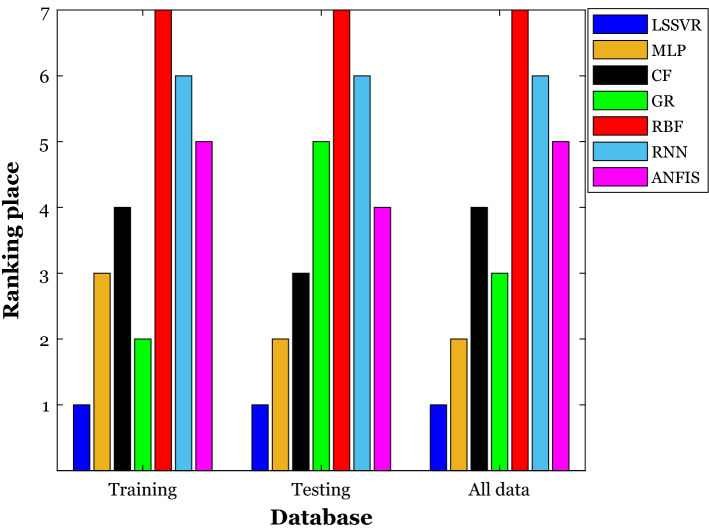
The ranking order of the intelligent estimators in different stages of the model development.

This ranking analysis has been separately applied to the model’s performances during the learning and testing stages. Furthermore, the rank orders of the chosen intelligent models have also been tracked over the whole 1239 datasets. Figure 1 displays the rank order of the LSSVR, artificial neural network models (i.e., MLP, RNN, RBF, CFF, and GR), and ANFIS over three different databases. It can be easily inferred that the LSSVR with the three first ranking places and the RBF neural network with the three seventh ranking places are the best and worst tools for calculating the density of deep eutectic solvents. The ranking order of other constructed models has also displayed in this figure.

In summary, it can be claimed that the LSSVR equipped with the Gaussian kernel function is the most trustful model for calculating the density of deep eutectic solvents from temperature and inherent characteristics (i.e., ω, Tc, and Pc) of the involved substance. This highly accurate model anticipates the density of 1239 deep eutectic solvents with the MAPE = 0.26%, MAE = 2.94, RAPE = 4.06%, RMSE = 5.65, and R^2^ = 0.99798.

### Validation stage

#### The LSSVR versus empirical correlations

**Figure 2:**
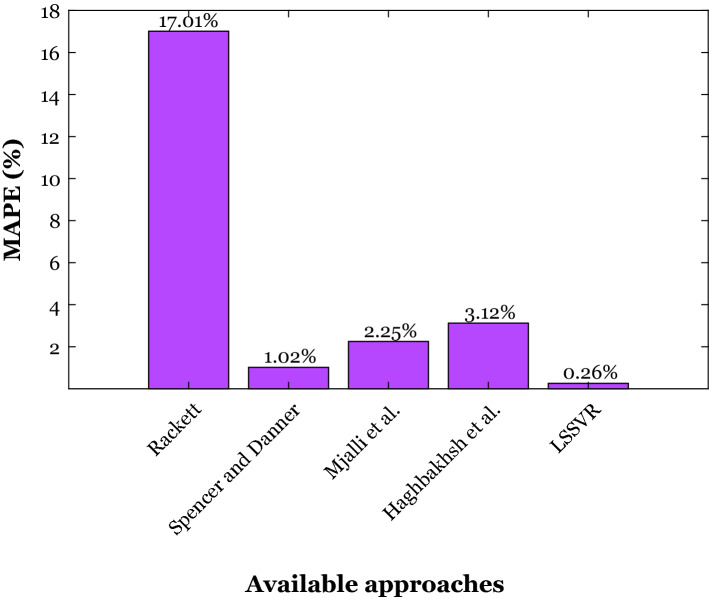
The prediction accuracy of the LSSVR and four empirical correlations in the literature^28^ to estimate the DES’s density of a completely similar database.

The accuracy of the suggested LSSVR and four empirical correlations in the literature (Rackett, Spencer and Danner, Mjalli et al., and Haghbakhsh et al.) for estimating 1239 densities of the deep eutectic solvent has been compared in the current section. The results of this comparison utilizing the MAPE have been described in Fig. 2. The observed results confirm that the LSSVR is the most accurate tool for estimating the density of deep eutectic solvents. The LSSVR anticipates 1239 density samples of 149 deep eutectic solvents with the MAPE = 0.26%, while the most accurate empirical correlation (Spencer and Danner model) presents the MAPE = 1.02% for an entirely similar database. The suggested LSSVR improves the best previously achieved accuracy by more than 74%.

#### Validation using graphical inspections

**Figure 3:**
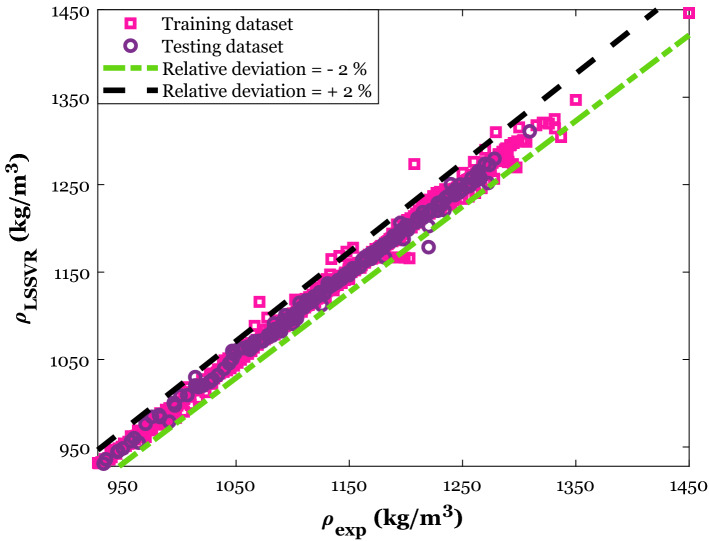
The consistency between experimental values of DES’s density and the LSSVR’s prediction.

The anticipated densities by the LSSVR ($\rho_{LSSVR}$) versus their counterpart experimental values (i.e., cross-plot) have been shown in Fig. 3. This cross-plot separately presents the LSSVR predictions for both learning and testing steps. Two straight lines associated with the relative deviation percent (RD%) of − 2% and + 2% have also been added to this figure. Equation (23) expresses the formula of the RD%.

23 $$RD\% = 100 \times \left[ {\left( {\rho_{\exp } - \rho_{LSSVR} } \right)/\rho_{\exp } } \right]_{i} \;\;\;i = 1,2, \ldots ,n$$

Figure 3 displays that about ten density samples have been anticipated with the RD% of lower than − 2% and higher than + 2%. The excellent ability of the built LSSVR to estimate the density of deep eutectic solvents can be readily approved by this observation.

**Figure 4:**
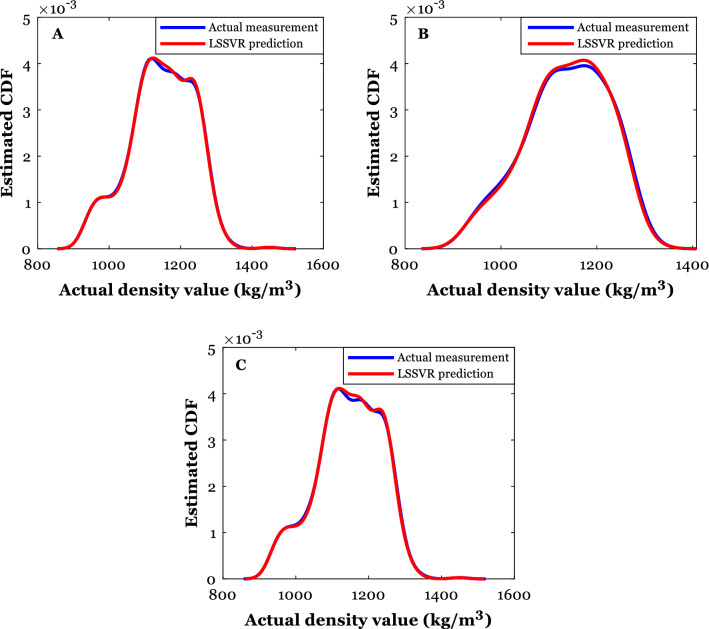
Utilizing the kernel density estimation method to check the LSSVR validity in the training (**A**) and testing (**B**) stages and against whole the databank (**C**).

The kernel density estimation is a reliable method for visually inspecting the compatibility between a given variable’s actual and anticipated values. As Fig. 4 shows, this method depicts the cumulative distribution function (CDF) as a function of the experimental values of a given variable. Figure 4A–C illustrate the compatibility between actual and anticipated density values over the training and testing subdivisions and the whole database. Excluding the intermediate values of the DES’s density, a remarkable consistency can be seen between actual and predicted values. Moreover, it can be detected that both the experimental data and the LSSVR predictions have a standard Gaussian distribution shape.

The magnitude of difference between actual and predicted densities (the residual error, i.e., RE) is another statistical index applied to monitor the prediction accuracy of the built LSSVER. The mathematical expression of the RE is given in Eq. (24).

24 $$RE_{i} = \left( {\rho_{\exp } - \rho_{LSSVR} } \right)_{i} \;\;\;i = 1,2, \ldots ,n$$

**Figure 5:**
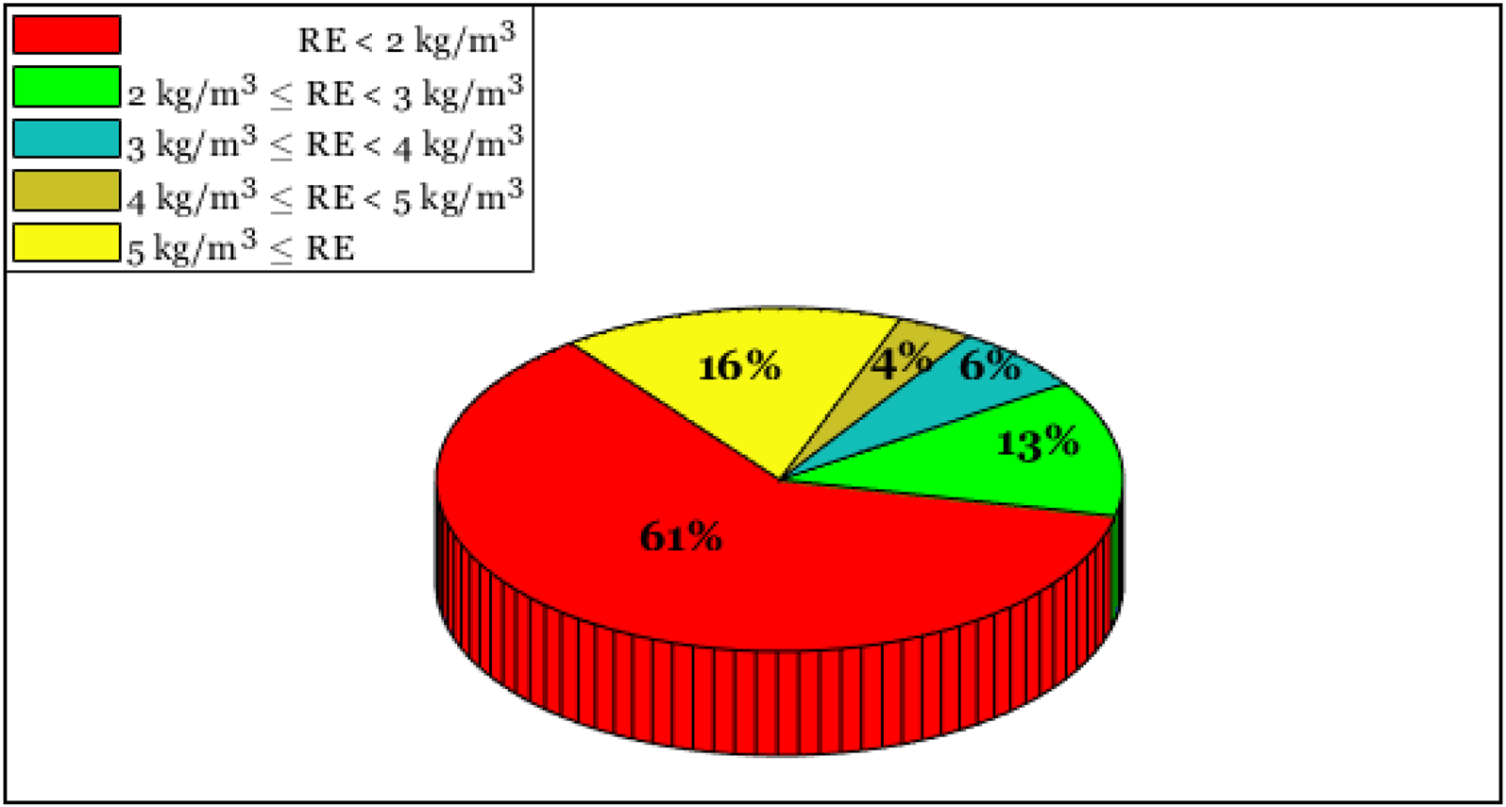
The cumulative frequency of the residual error (RE) of the LSSVR for estimating the DES’s density.

Based on reported results in Fig. 5, 61% of the available samples have been estimated with a residual error of less than 2 kg/m^3^. Moreover, the LSSVR successfully anticipated 84% of the experimental databank with an RE of lower than 5 kg/m^3^. Only 16% of the gathered database has been estimated with a residual error of higher than 5 kg/m^3^. All these observations confirm the excellent compatibility between calculated densities by the LSSVR and their related actual measurements.

### Checking the reliability of the gathered database

The gathered experimental data had a central role during the development/validation/selection of machine learning methods hereinbefore. Furthermore, this experimental databank has been used to compare the accuracy of empirical correlations and the selected LSSVR. The entire previous findings are valid only if the gathered laboratory-measured densities have an acceptable validity level. The leverage is a well-trusted technique to detect both valid and outlier data in an experimentally-measured database^94^. This technique plots the standardized residuals (*SR*) against the Hat index to accomplish its duty^89^. Equation (27) explains that the *SR* can be obtained by dividing the average value ($RE^{ave}$) and standard deviation (*SD*) of the residual error. Equations (25) and (26) give the $RE^{ave}$ and *SD* formula, respectively.

25 $$RE^{ave} = \left( {1/n} \right) \times \sum\limits_{i = 1}^{n} {RE_{i} }$$

26 $$SD = \sqrt {\sum\limits_{i = 1}^{n} {\left( {RE_{i} - RE^{ave} } \right)}^{2} /n}$$

27 $$SR_{i} = RE_{i} /SD\;\;i = 1,2,...,n$$

Furthermore, numerical values of the Hat index (*HI*) can be reached by applying Eq. (28) on the matrix of the independent variables ($\xi$)^95^. The superscripts of *T* and *-1* stand for the transpose and inverse operations, respectively.

28 $$HI = \xi \left( {\xi^{T} \xi } \right)\xi^{ - 1}$$

**Figure 6:**
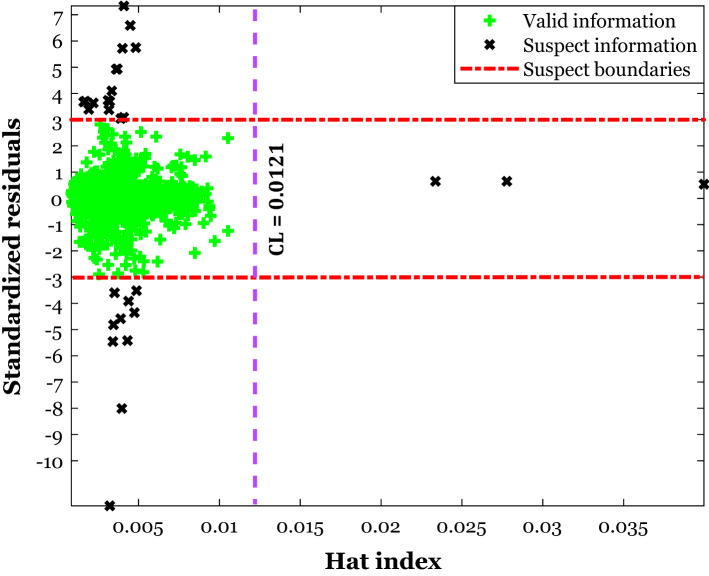
The results of applying the leverage method on the gathered density databank.

Figure 6 shows the plot of *SR* versus the *HI* values associated with the DES’s density databank. The leverage method states that the region bounded by the -3 < *SR* <  + 3 and *HI* lower than the critical leverage is valid, and all other positions are the suspect domain^96^. Equation (29) helps calculate the critical leverage (*CL*) from the number of independent variables (*NIV*) and experimental data points (*n*)^83,95^. Having four independent variables and 1239 data points, the *CL* equals 0.0121.

29 $$CL = 3 \times \left( {NIV + 1} \right)/n$$

The leverage method approves that 1210 out of 1239 data points have appeared in the valid zone, and only 29 density samples may be outlier measurements. It can be claimed that the validity of the gathered database has been approved now, and all previous findings based on this databank are trustful.

### LSSVR accuracy for density predicting each deep eutectic solvent

It may be a good idea to monitor the prediction accuracy of the LSSVR against the deep eutectic solvents with the same HBA agent. Since the average relative deviation (Eq. 30)^97^ clarifies both underestimated and overestimated predictions, it has been selected to measure the LSSVR accuracy in this stage.

30 $$ARD\% = \left( {100/n} \right) \times \sum\limits_{i = 1}^{n} {\left[ {\left( {\rho_{\exp } - \rho_{LSSVR} } \right)/\rho_{\exp } } \right]}_{i}$$

**Figure 7:**
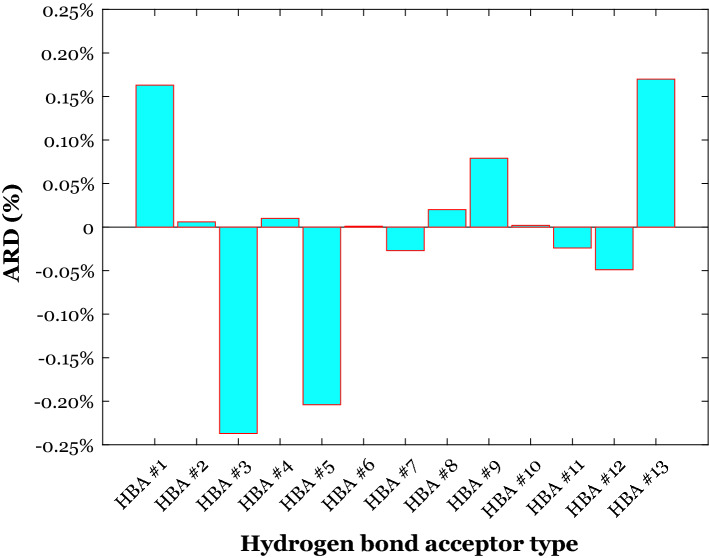
The observed average relative deviation between actual and predicted densities of the deep eutectic solvent with the same HBA agent.

Figure 7 states that the density of thirteen classes of the deep eutectic solvent with the HBA#1 to HBA#13 (see Table 1) has been estimated with the ARD ranges from − 0.24 to + 0.17%. Those deep eutectic solvents having the HBA #1, 9, and 13 have been underestimated by the LSSVR. On the other hand, the DESs with the HBA #3, 5, and 12 have been overestimated. The ARD% associated with the other deep eutectic solvent classes is almost equal to zero.

### Investigating the effect of temperature, and HBD/HBA types

**Figure 8:**
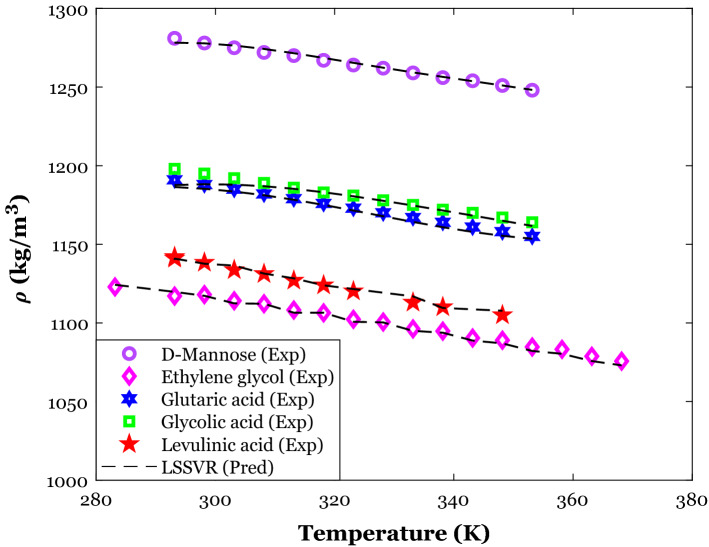
The excellent performance of the LSSVR model for correctly identifying the HBD effect on the density of Choline chloride as an HBA.

The effect of temperature on the density of deep eutectic solvents with the specific HBA agent (i.e., Choline chloride) and different HBD substances can be deduced from Fig. 8. This figure reports both experimentally-measured densities and their counterparts simulated values by the LSSVR. This figure readily justifies an excellent agreement between experimental and predicted density values. The LSSVR effectively discriminates between the effect of HBD type and working temperature on the density of the Choline chloride-based DESs and accurately estimates all distinct data points. Like the conventional liquid, the density of deep eutectic solvents decreases by increasing the working temperature. Increasing the intermolecular void volume in the DES’s body by increasing the temperature has been introduced as responsible for this observation^98^.

**Figure 9:**
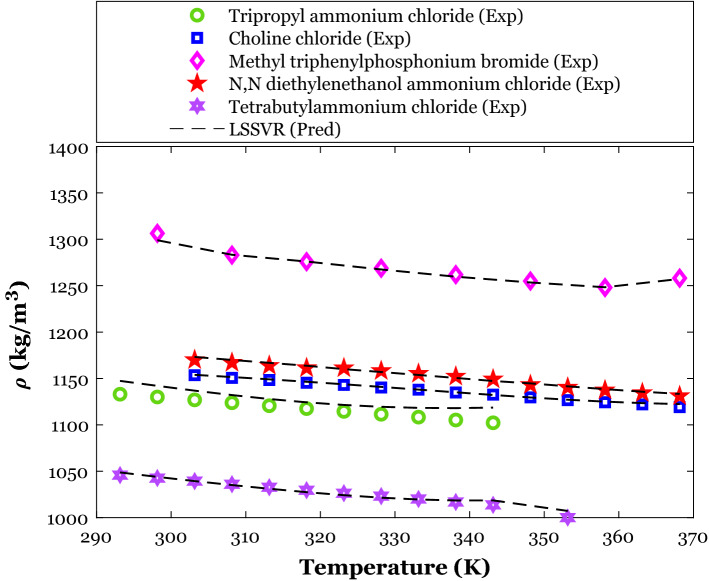
Monitoring the ability of the LSSVR model to anticipate the HBA effect on the density of the glycerol as an HBD.

The density variation of deep eutectic solvents with the temperature and HBA type has been exhibited in Fig. 9. All DESs in this analysis have glycerol as their HBD agent. A high level of compatibility between actual density values and their counterparts estimated by the LSSVR can be seen in Fig. 9. The LSSVR distinguishes the effect of HBA type and temperature on the DES’s density and accurately anticipates all individual density data points.

## Simple flowchart of our study

**Figure 10:**
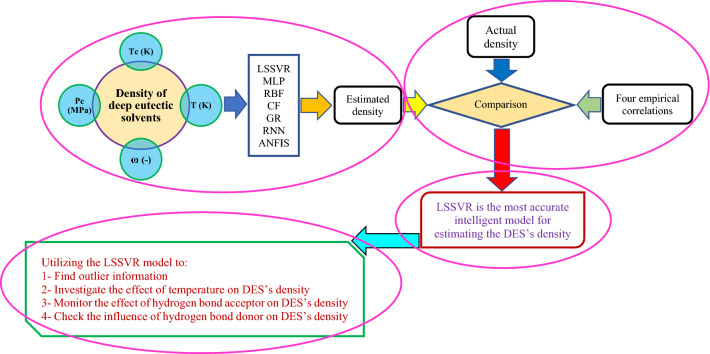
A simple flowchart for explaining the stages followed in the present study.

A simple and understandable flowchart for the stages followed in the current research study has been presented in Fig. 10. This figure can be broken down into four distinct parts as follows:

1. Developing machine learning methods
2. Comparing accuracy performances of the machine learning methods and empirical correlations
3. Selecting the model with the highest prediction accuracy
4. Utilizing the model chosen for further analyzing purposes

## Conclusion

The accuracy of seven machine learning methods and four empirical correlations has been compared to find the highest accurate tool for estimating the density of 149 deep eutectic solvents. Huge performed statistical analyses proved that the least-squares support vector regression equipped with the Gaussian kernel function is more accurate than the other methods investigated. This suggested scheme predicted 1239 experimentally-measured densities with the MAPE = 0.26%, MAE = 2.94, RAPE = 4.06%, RMSE = 5.65, and R^2^ = 0.99798. Visual inspections utilizing the cross-plot, kernel density estimation, residual error, and average relative deviation have also justified a high level of compatibility between LSSVR predictions and their experimentally-measured counterparts. Investigating the experimental database employing the leverage technique resulted in founding 1210 valid and 29 suspect information. Furthermore, the fabricated LSSVR successfully infers the effect of temperature and HBA and HBD types on the density of the deep eutectic solvent. The current research may be viewed as an initiative towards constructing reliable models for anticipating DESs properties. Such a model promotes an efficient solvent synthesis that can help design and simulate new processes utilizing the DES.

## Supplementary Information

Supplementary Information.

## Data Availability

All data generated or analyzed during this study are included in the supplementary file.
